# How active are people in metropolitan parks? An observational study of park visitation in Australia

**DOI:** 10.1186/s12889-015-1960-6

**Published:** 2015-07-04

**Authors:** Jenny Veitch, Alison Carver, Gavin Abbott, Billie Giles-Corti, Anna Timperio, Jo Salmon

**Affiliations:** Centre for Physical Activity and Nutrition Research, Deakin University, 221 Burwood Highway, Burwood, Victoria 3125 Australia; Melbourne School of Population and Global Health, The University of Melbourne, Melbourne, Australia

**Keywords:** Observation, Park, Physical activity, Public open space, Park visitor

## Abstract

**Background:**

Parks are generally an under-utilized resource in the community with great potential to enhance levels of physical activity. If parks are to attract more visitors across a broad cross-section of the population and facilitate increased physical activity, research is needed to better understand park visitor characteristics and how visitors spend their time in parks. The Recording and EValuating Activity in a Modified Park (REVAMP) study is a natural experiment monitoring a park upgrade in a low socioeconomic status (SES) neighborhood. This study described the observed baseline characteristics of park visitors (age, sex) and characteristics of visitation (weekday or weekend day, period of the day) and explored how these characteristics were associated with observed park-based physical activity in two metropolitan parks located Melbourne, Australia.

**Methods:**

Direct observations of park visitors were conducted using a modified version of SOPARC (the System for Observing Play and Recreation in Communities) on four weekdays and four weekend days. During weekdays, observations were conducted every hour from 7:30 am-4:30 pm and on weekend days from 8:30 am-4:30 pm. This equated to a total of 1460 scans across the two parks. Chi-square tests examined bivariate associations between park-based physical activity, and socio-demographic and park visitation characteristics. Logistic regression models examined the odds of being observed engaging in moderate- to vigorous-intensity physical activity relative to lying/sitting/standing according to socio-demographic and park visitation characteristics.

**Results:**

In total, 4756 park visitors were observed with the majority visiting on weekend days (87 %) and in the afternoon (41 %). Most visitors (62 %) were lying, sitting or standing, with only 29 % observed engaging in moderate-intensity and 9 % in vigorous-intensity physical activity. Park use differed by time of day, sex, age group, and neighborhood SES. Physical activity was lower for women than men (OR 0.76) and higher among visitors in the high SES area (OR 1.52).

**Conclusions:**

Parks offer substantial opportunities for people of all ages to engage in physical activity; however, this study showed that a large proportion of the park visitors observed were engaged in sedentary pursuits. Further research on how park design, amenities and programming can optimize park visitation and park-based physical activity is needed.

**Trial Registration:**

Current controlled trial ISRCTN50745547, registration date 11.1.2014

## Background

Public open spaces or parks are located in most neighborhoods in developed countries, they are generally free to access, offer a variety of opportunities for physical activity and can serve diverse populations [1]. Parks may encourage physical activity in two ways: as a destination to which people walk or cycle (i.e. active transport) and as a setting in which physical activity can take place [2, 3]. Both of these ‘opportunities’ for physical activity may make substantial contributions to overall physical activity levels and therefore benefit public health. Research shows that park availability, proximity and access are associated with higher overall levels of physical activity [4]. A number of studies have found park quality and specific park features to be major factors associated with park-based physical activity [3, 5–7]. Despite the significant potential for parks to promote physical activity, parks are generally under-utilized [8], and efforts to increase use could potentially augment current physical activity levels.

A growing body of literature documents differences in park-based physical activity across various park facilities and settings. Identifying physical activity levels and time spent sedentary (sitting) among visitors in parks and how this varies by age, sex, and time of the day can inform the development of strategies to promote park-based physical activity among those insufficiently active. One way to evaluate park usage and park-based physical activity is through direct observations of park visitors. Observational studies of park visitation in the United States (US) have shown that more than half of park visitors engaged in sedentary behavior (primarily sitting) during their park visit [8–12]. They have also shown that most park visitors are male and that adults and children were more likely to visit parks than adolescents and seniors. More males and children were observed to be active in parks than females and adults [8, 12, 13].

Fewer observation studies of park visitors have been conducted in Australia and they have been primarily limited to small neighborhood parks [14, 15] or focused only on children [16]. However, all of these studies were designed to examine the impact of park refurbishment on park visitation and did not report park visitor characteristics or how these characteristics were associated with park visitation.

In Australia, metropolitan parks are large parks that provide informal and/or organised recreation for large numbers of people associated with enjoyment of natural or semi-natural surroundings or open space. They are designed to attract visitors beyond the immediate surrounding neighborhoods to engage in a variety of physical and social activities. Due to their size and range of facilities and amenities, metropolitan parks offer significant opportunities for physical activity; however, to our knowledge no previous systematic observations of visitors of metropolitan parks have been conducted in Australia. In addition, few studies have examined how usage varies between parks located in high versus low socio-economic status (SES) neighborhood areas [17–19] or how park usage varies on weekdays compared with weekend days or according to the period of the day (i.e. morning versus afternoon) [20–22]. This type of information is important because it provides insights into how these metropolitan parks are used, and their potential to further promote physical activity.

In summary, parks are generally an under-utilized resource in the community with great potential to enhance levels of physical activity. If parks are to attract more visitors across a broad cross-section of the population and facilitate increased physical activity, research is needed to better understand park visitor characteristics and how visitors spend their time in parks. The purpose of this study was to describe the socio-demographic characteristics of park visitors (age, sex) and characteristics of visitation (weekday or weekend day, period of the day) and to explore how these characteristics are associated with observed park-based physical activity in two metropolitan parks located in high and low SES areas of Melbourne, Australia.

## Methods

For this study, baseline observational data from the Recording and EValuating Activity in a Modified Park (REVAMP) study were used. Details on the study protocol have been published elsewhere [23]. Briefly, the REVAMP study is a natural experiment that aims to examine whether park improvements (installation of a new playground for children of all abilities) increases overall park usage, park-based physical activity and active travel to and from the park in an intervention park compared with a control park over a two-year period; and to identify which specific aspects of the park refurbishment attract park visitors and encourage park visitors to be more active. The study protocol was approved by the Deakin University Human Research Ethics Committee, the Victorian Department of Education and Early Childhood Development and the Catholic Education Office.

Baseline measurements were conducted in April-May (Autumn) 2013, prior to the intervention park being refurbished, at two large metropolitan parks in Melbourne, Australia. There are 15 metropolitan parks located in Melbourne ranging in size from 38-1064 ha (mean size 334 ha). The intervention park (329 ha) is located 28 km (17 miles) north-west of Melbourne’s central business district (CBD) in a low SES area according to the Victorian Socio-Economic Index for Areas (SEIFA) distribution [24]; and the control park (120 ha) is located 22 km (13 miles) east of Melbourne’s CBD in a high SES area. The local government area within which the intervention park is located (City of Brimbank) has a total population of almost 190,000 residents with a high proportion of children aged 0–9 years (12.7 %), a growing indigenous population (currently 0.4 %), and a high proportion of residents born overseas (49.6 %). The local government area within which the control park is located (City of Manningham) has a total population of 111,000 residents, 10.4 % are aged 0-9 years, 0.1 % are indigenous, and 36.5 % are born overseas [25]. Despite the differences in overall size and SES area, at baseline these two parks had similar features that provided opportunities to be active such as extensive walking/cycling paths, grassy open space areas, trees, and basic playground equipment. The parks also provided other supportive amenities that may encourage visitation such as a café, a variety of picnic shelters and tables, barbeque areas, toilets and car-parking (see Table 1).

**Table 1:** Summary of park facilities/amenities

Park facilities/amenities	Park located in low SES area	Park located in high SES area
Outdoor sports courts (i.e. tennis, basketball)	No	No
Sports ovals	No	No
Number of playgrounds	2	1
Shade over playgrounds	No	No
Separate playground areas for different age groups	No	No
Toilets	Yes	Yes
Kiosk	Yes	Yes
Drinking fountains	Yes	No
Shelters, Picnic tables, benches, bbq’s	Yes	Yes
Bike racks	Yes	No
Rubbish bins	No	No
Litter present	Very little	Some
Risky litter visible	No	Very little
Graffiti visible	No	No
Water feature (i.e. River, creek)	No	Yes
Trees	Yes	Yes
Signs saying dogs must be on leash	Yes	Yes
Dog litter bags provided	No	No
Paths or cycleways within the park	Yes	Yes
Designated cycle path/trail link to park	Yes	Yes
Car parking facilities within the park	Yes	Yes
Access to public transport within one block of park	Yes	Yes
Pedestrian crossing on bordering streets to assist access to the park	No	No

### Observations

To assess park usage and park-based physical activity, direct observations of park visitors were conducted using a modified version of SOPARC (the System for Observing Play and Recreation in Communities) [13]. This is a reliable, objective observation tool for assessing physical activity in community settings and previous studies have used SOPARC to specifically assess physical activity in parks [20, 26]. This instrument is based on momentary time sampling and involves undertaking systematic scans (an observation sweep moving from left to right) of each participant within a target area at a particular time.

At each park, six trained observers conducted observations of ten clearly defined target areas. Target areas were pre-determined after discussions with the park rangers to determine the most highly visited areas and included, for example, the playground, walking/cycling paths, grassy open spaces, shelters and picnic areas. During each scan, trained, clearly identifiable research assistants recorded each individual in view within their target area according to: their broad age group (i.e., child [1-12 years], teen [13-20 years], adult [21-59 years], or older adult [60 years+]); sex (male or female); and the activity they were engaged in (i.e., lying down or sitting, standing, moderate activity, or vigorous activity). Data were collected for a total of eight days, including four weekdays and four weekend days. During weekdays, observations were conducted every hour from 7:30 am-4:30 pm (except for one day when observations concluded at 1:30 pm due to rain), and on weekend days every hour from 8:30 am-4:30 pm. The actual days and times of data collection were the same for both parks. This equated to a total of 1460 target area scans across the two parks (2 parks * 10 target areas * 73 time points). For this study, observations conducted at 7.30 am and 8.30 am were classified as morning; 9.30 am, 10.30 am and 11.30 am as mid-morning; 12.30 pm and 1.30 pm as midday; and 2.30 pm, 3.30 pm and 4.30 pm as afternoon. Observations were not conducted on days of forecasted rain. The average temperature during the observation days was 11.8 °C (minimum) and 19.5 °C (maximum), with sunrise approximately 6.50 am and sunset 5.45 pm.

### Training of observers

Training of observers was conducted during a classroom workshop and on-site park visits where trainees practiced recording park visitors and then received feedback on their coding. After training was complete, inter-observer agreement was tested with observers completing 12 observations at the intervention park and 20 observations at the control park. Across all reliability observations, 92 % had at least 80 % agreement between the observers and 86 % of scans had 100 % agreement, indicating strong inter-rater reliability for all recorded visitor characteristics.

### Analysis

Descriptive statistics were used to describe the park visitor characteristics. Chi-square tests were conducted to examine bivariate associations between park-based sitting, standing, moderate and vigorous-intensity physical activity, and socio-demographic and park visitation characteristics. Logistic regression models were conducted to examine the odds of being observed engaging in moderate- to vigorous-intensity physical activity (MVPA) relative to lying/sitting/standing (reference category) according to socio-demographic and park visitation characteristics. All analyses were conducted using StataSe v12 (StataCorp, TX) and findings were considered significant at p < 0.05.

## Results

Across the eight observation days, a total of 4756 visitors were observed in the two parks with an almost equal number observed at each park (see Table 2). Males and females were almost equally split, and approximately half of all visitors were adults (21-59 years, 53 %). The majority were observed on weekend days (87 %) and in the afternoon (41 %). Twenty five percent were observed lying/sitting, 37 % were observed standing, 29 % in moderate-intensity and 9 % in vigorous-intensity physical activity.

**Table 2:** Park visitor characteristics

Park visitor characteristics	n (total n = 4756)	Percent
Sex		
Male	2316	48.7
Female	2440	51.3
Age		
Child (1-12 years)	1112	23.4
Teen (13-20 years)	353	7.4
Adult (21-59 years)	2542	53.4
Senior (60+ years)	749	15.7
Physical activity level		
Lying/sitting	1168	24.6
Standing	1771	37.2
Moderate	1371	28.8
Vigorous	446	9.4
Day of week		
Weekday	819	17.2
Weekend day	3937	87.3
Park		
Low SES	2374	49.9
High SES	2382	50.1
Period of day		
Morning^a^	137	2.9
Mid-morning^a^	1223	25.7
Midday^a^	1445	30.4
Afternoon^a^	1951	41.0

^a^Morning included 2 observation periods on weekdays (7.30 am & 8.30 am) and 1 observation period on weekend days (8.30 am); mid-morning included 3 observation periods (9.30 am, 10.30 am & 11.30 am), midday included 2 observation periods (12.30 pm & 1.30 pm), afternoon included 3 observation periods (2.30 pm, 3.30 pm & 4.30 pm)

The characteristics of park visitation according to age groups are shown in Table 3. Significant differences in park usage according to age groups were observed for day of the week, park attended and period of the day. A higher proportion of teens were observed on weekdays compared with weekend days and more adults were observed on weekend days than weekdays. A higher proportion of children were observed at the high rather than low SES area park. No children were observed in the morning, more teens were observed in the morning than at other times of the day, and more adults and fewer seniors were observed mid-morning compared with other times of the day. There were no significant differences in the proportions of male or female visitors observed by age group.

**Table 3:** Associations between observed socio-demographic park visitation characteristics and the four age groups

	Child (n = 1112)	Teen (n = 353)	Adult (n = 2542)	Senior (n = 749)	*χ* ^2^ (df)	Cramer’s V	p
Sex n (%)							
Male	572 (24.7)	174 (7.5)	1196 (51.6)	374 (16.2)	6.616 (3)	0.037	0.085
Female	540 (22.1)	179 (7.3)	1346 (55.2)	375 (15.4)			
Day of week n (%)							
Weekday	183 (22.3)	194 (23.7)	316 (38.68)	126 (15.4)	394.092 (3)	0.288	**<0.0005**
Weekend day	929 (23.6)	159 (4.0)	2226 (56.5)	623 (15.8)			
Park n (%)							
Low SES	434 (18.3)	188 (7.9)	1325 (55.8)	427 (18.0)	74.333 (3)	0.125	**<0.0005**
High SES	678 (28.5)	165 (6.9)	1217 (51.1)	322 (13.5)			
Period of day n (%)							
Morning	0	36 (26.3)	72 (52.6)	29 (21.2)	185.003 (9)	0.114	**<0.0005**
Mid-morning	232 (19.0)	116 (9.5)	744 (60.8)	131 (10.7)			
Midday	387 (26.8)	75 (5.2)	751 (51.9)	232 (16.1)			
Afternoon	493 (25.3)	126 (6.5)	975 (50.0)	357 (18.3)			

Chi-square tests of independence used to compare park visitor usage according to observed age group

P: bolded indicates significant associations

The characteristics of park visitation according to activity level are shown in Table 4. A higher proportion of females than males were observed sitting. Compared with the other age groups, a lower proportion of children were observed standing and a higher proportion observed in moderate and vigorous-intensity physical activity. Fewer seniors were observed in vigorous activity. A higher proportion of people were observed engaged in vigorous activity on weekend than weekdays, and at the high SES park compared with the low SES park. In the mornings, no-one was observed sitting and a higher proportion of people were observed in moderate- or vigorous-intensity physical activity compared with other times of the day.

**Table 4:** Associations between observed socio-demographic and park visitation characteristics and physical activity

	Sitting (n = 1168)	Standing (n = 1771)	Moderate (n = 1371)	Vigorous (n = 446)	*χ* ^2^ (df)	Cramer’s V	p
Sex n (%)							
Male	455 (19.7)	901 (38.9)	702 (30.3)	258 (11.1)	66.125 (3)	0.118	**<0.0005**
Female	713 (29.2)	870 (35.7)	669 (27.4)	188 (7.7)			
Age group n (%)							
Child	244 (21.9)	235 (21.1)	448 (40.3)	185 (16.6)	303.000 (9)	0.146	**<0.0005**
Teen	71 (20.1)	168 (47.6)	82 (23.2)	32 (9.1)			
Adult	621 (24.4)	1079 (42.5)	628 (24.7)	214 (8.4)			
Senior	232 (30.9)	289 (38.6)	213 (28.4)	15 (2.0)			
Day of week n (%)							
Weekday	189 (23.1)	314 (38.3)	267 (32.6)	49 (5.9)	18.254 (3)	0.062	**<0.0005**
Weekend day	979 (24.9)	1457 (37.0)	1104 (28.0)	397 (10.1)			
Park n (%)							
Low SES	615 (25.9)	970 (40.9)	633 (26.7)	156 (6.6)	67.707 (3)	0.119	**<0.0005**
High SES	553 (23.2)	801 (33.6)	738 (30.9)	290 (12.2)			
Period of day n (%)							
Morning	0	22 (16.1)	78 (56.9)	37 (27.0)	272.829 (9)	0.138	**<0.0005**
Mid-morning	219 (17.9)	397 (32.5)	450 (36.8)	157 (12.8)			
Midday	394 (27.3)	623 (43.1)	324 (22.4)	104 (7.2)			
Afternoon	555 (28.4)	729 (37.4)	519 (26.6)	148 (7.6)			

Chi-square tests of independence used to compare park visitor usage according to observed activity level

P: bolded indicates significant associations

Logistic regression results are presented in Table 5 and show that females had 24 % lower odds than males of being observed in MVPA compared with sitting or standing. Compared with children, the other three age groups all had lower odds of being observed engaging in MVPA. Compared with the low SES park, visitors in the high SES area park had 52 % greater odds of being observed engaging in MVPA. Park visitors in the mid-morning, midday and afternoon periods had lower odds of engaging in MVPA compared with visitors in the morning period. There were no differences in the odds of being observed engaging in MVPA between weekdays and weekend days.

**Table 5:** Odds ratios for associations between observed socio-demographic and park visitation characteristics and MVPA

	Sitting/standing (ref) vs MVPA	
	OR (95 % CI)	p
Sex		
Male (ref)	1.00	
Female	0.76 (0.68, 0.86)	**<0.0005**
Age group		
Child (ref)	1.00	
Teen	0.36 (0.28, 0.46)	**<0.0005**
Adult	0.37 (0.32, 0.43)	**<0.0005**
Senior	0.33 (0.27, 0.40)	**<0.0005**
Day of week		
Weekday (ref)	1.00	
Weekend day	0.98 (0.84, 1.14)	0.806
Park		
Low SES (ref)	1.00	
High SES	1.52 (1.36, 1.72)	**<0.0005**
Period of day		
Morning (ref)	1.00	
Mid-morning	0.19 (0.12, 0.30)	**<0.0005**
Midday	0.08 (0.05, 0.13)	**<0.0005**
Afternoon	0.09 (0.09, 0.16)	**<0.0005**

OR = Odds Ratios; 95 % CI = 95 % confidence interval

P: bolded indicates significant associations

## Discussion

This study describes the characteristics and physical activity levels of park visitors in two large metropolitan parks in Melbourne, Australia. Given that physical inactivity is a major contributor to the burden of chronic disease, including cardiovascular disease, diabetes, and overweight and obesity [27], understanding the characteristics of park visitors and park visitation is important in order to develop strategies to increase park use and park-based physical activity.

Across all observations, 62 % of park visitors were observed lying, sitting or standing. This is an important observation and shows that visiting large metropolitan parks is not always for physical activity purposes. This is consistent with observational studies of park visitation in the US reporting that more than half of park visitors engage in sedentary behavior (primarily sitting) during their park visit [9, 12]. A previous Australian study found that compared with poorly designed parks, more people used attractive parks, and that visitors engaged in a wider range of activities, including sedentary behaviors such as picnics and sitting [5]. The parks included in the current study are large attractive metropolitan parks with amenities that attract visitors from outside the neighborhood and which encourage picnics and social gatherings. These activities are more likely to involve sedentary behaviors. Previous research has shown that visitors who travel greater distances to a park tend to visit for picnics and sedentary social activities with family [28]. Although spending time in parks may benefit mental health [29], as such a large percentage of park visitors were engaging in low levels of physical activity, it is important to better understand how to encourage park users to engage in physical activity while they are in the park. Simply offering attractive large parks may be insufficient to increase already low community-wide physical activity levels.

Some between-park differences in park visitation were observed. More children and seniors were observed in the park located in the low SES area compared with the park in the high SES area and almost seven in ten park visitors were observed lying, sitting or standing in the park in the low SES area, compared with only 57 % of visitors engaging in these behaviors in the park in the high SES area. In addition, park visitors at the park in the high SES area had greater odds of engaging in MVPA and a higher proportion were observed engaged in vigorous activity compared with park visitors in the low SES area. This is in contrast to previous research that showed visitors of low-income neighborhood parks to be more vigorously active than visitors of high-income neighborhood parks [30], although the overall park activity-related intensity in that study did not differ between high- and low-income neighborhoods.

It is possible that the differences in park visitation observed in the current study between the two parks may be reflected by different demographic profiles of residents living near the two parks. Considering that residents living in low SES areas are more likely to be at an increased risk of inactivity and associated poor health outcomes [31], it is important to understand why there may be differences in park-based physical activity between visitors to parks in a low and a high SES area. Previous research in Melbourne, Australia found that parks in low SES areas had fewer amenities and features likely to promote physical activity among children than parks in higher SES areas [32]. Although it is acknowledged that the data used in the current study were collected prior to the development of a new playground at the park in the low SES area, the comparison park in the high SES area was selected based on having similar features and amenities to that of the intervention park at baseline. It is therefore possible that the lower level of MVPA observed in the park in the low SES area may be due to cultural or social factors as the demographic profile of residents in this area is diverse with a growing indigenous population and a high proportion of residents born overseas (49.6 %) [25]. Longitudinal research has shown that having access to green space near home may not be adequate for initiating regular walking habits; however, it seems to be important for maintenance of walking habits [33]. Additional factors such as within park programming may be required to stimulate adults to take up walking or other forms of physical activity in parks. This may be particularly important in low SES areas where visitors may be less likely to be physically active when visiting the park.

The majority of park visitors in the current study were observed on weekend days, but there were no differences in the odds of being observed engaging in MVPA between weekdays and weekend days. Time of day did appear to be important; however, as park visitors were more physically active in the mornings. Previous studies have also found the morning to be a key period for park-based physical activity, with fewer visitors observed being sedentary and more visitors observed walking [30] and engaged in vigorous activity [20] in the morning compared with other times of the day. Thus, it may be important for park programmers and future intervention studies to consider the time of the day and maximise opportunities in the morning when park visitors are most likely to be active, as well as developing strategies to increase activity in the other less active periods of the day. Females were less likely than males to be observed in MVPA, so future research may also consider exploring opportunities for women to engage in park-based physical activity.

It is important to acknowledge the study limitations. Firstly, studies involving direct observations provide contextually-rich information about settings in which physical activity occurs; however, they are a snapshot in time that provide a general indication of park visitation on specified days and demographic characteristics, such as age, are estimated based on best guess. Additional methodological limitations include the cross-sectional design, inclusion of only two parks, and the completion of observations in the one season which limits the ability to generalize the results to other parks and across seasons. It is also important to acknowledge some inconsistencies in the number of observation scans included in the coding of periods of the day. For example, the midday period included two scan periods (12.30 pm and 1.30 pm) whereas the afternoon period included three scan periods (2.30 pm, 3.30 pm and 4.30 pm). Despite these limitations, the study involved a large number of observations which were conducted from early morning until late afternoon on both weekdays and weekend days, the reliability data collected showed high inter-observer agreement which provides adequate confidence in the measures used, and there was consistency in the measures with observations completed at the same time and day at both parks.

## Conclusions

Parks offer substantial opportunities for people of all ages to engage in physical activity; however, this study showed that a large proportion of the park visitors observed were engaged in sedentary pursuits. Differences in park visitation were observed according to the sex of the park visitor, age group, the period of the day, and the SES of neighborhood within which the park was located. Further research is needed to examine how usage varies in parks with different features and amenities and how programming and park design can optimize park visitation and park-based physical activity for all park visitors.
